# Extracellular vesicles and melatonin benefit embryonic develop by regulating reactive oxygen species and 5‐methylcytosine

**DOI:** 10.1111/jpi.12635

**Published:** 2020-02-16

**Authors:** Pengxiang Qu, Shiwei Luo, Yue Du, Yanru Zhang, Xiaojie Song, Xuetao Yuan, Zujie Lin, Yuchen Li, Enqi Liu

**Affiliations:** ^1^ Laboratory Animal Centre Xi’an Jiaotong University Health Science Centre Xi’an Shaanxi China; ^2^ NDCLS Radcliff Department of Medicine University of Oxford Oxford UK

**Keywords:** 5‐methylcytosine, embryo, extracellular vesicles, melatonin, oviduct fluid, reactive oxygen species

## Abstract

Embryo culture conditions are crucial as they can affect embryo quality and even offspring. Oviductal extracellular vesicles (EVs) long been considered a major factor influencing interactions between the oviduct and embryos, and thus its absence is associated with inferior embryonic development in in vitro culture. Herein, we demonstrated that melatonin is present in oviduct fluids and oviduct fluid‐derived EVs. Addition of either EVs (1.87 × 10^11^ particles/mL) or melatonin (340 ng/mL) led to a significant downregulation of reactive oxygen species (ROS) and 5‐methylcytosine (5‐mC), as well as an increase in the blastocyst rate of embryos, which was inhibited by the addition of luzindole—a melatonin receptor agonist. A combination of EVs (1.87 × 10^10^ particles/mL) and melatonin (at 34.3 pg/mL) led to the same results as well as a significant decrease in the apoptosis index and increase in the inner cell mass (ICM)/trophectoderm (TE) index. These results suggest that an EV‐melatonin treatment benefits embryonic development. Our findings provide insights into the role of EVs and melatonin during cell communication and provide new evidence of the communication between embryos and maternal oviduct.

## 1 INTRODUCTION

Significant progress has been made in assisted reproduction techniques (ART) for the treatment of infertility ever since Louise Brown, the first test‐tube baby, was successfully conceived by in vitro fertilization (IVF) in 1978.^1^ During the past 40 years, the number of infants conceived through IVF accounts for approximately 0.1% of the total global birth population.^2^ This number is predicted to reach around 3.5% of the total birth population by 2100.^2^ Many factors affect embryonic development in vitro, which determines the efficiency of ART, especially IVF, intracytoplasmic sperm injection, and cryopreservation.^3^ Culture media are indispensable for ART, which commonly consist of inorganic salts, carbohydrates, amino acids, vitamins, nucleic acid precursors, chelating agents, antioxidants, antibiotics, macromolecules, hormones, and growth factors.^4^ Although the composition of the culture media is being continuously optimized, embryo development in vitro is still poor regarding embryo metabolism, morphology, epigenetic changes, and associated gene expression.^5^, ^6^ Moreover, the culture media may exert a long‐term detrimental effect on offspring after birth.^7^ Therefore, there is an urgent need to optimize the culture media of embryos to improve ART efficiency.

Melatonin is a ubiquitous molecule present in almost every organism from bacteria to humans.^8^, ^9^ It is involved in sleep and circadian rhythms, antioxidant activity, and immunity enhancement.^10^ In addition, melatonin is regarded as a multifunctional oncostatic agent^11^, ^12^ and is potentially therapeutic for nonsmall cell lung cancer,^13^ colorectal cancer,^14^ Alzheimer's disease,^15^ chronic kidney disease,^16^ and acute ischemia‐reperfusion liver injury.^17^ Notably, some studies have showed that melatonin is involved in reproduction, fertility, and development.^18^, ^19^, ^20^

Extracellular vesicles (EVs) have been reported to mediate intercellular interactions by transferring noncoding RNAs and proteins.^21^ Furthermore, oviductal EVs play an important role in oviduct–embryo interactions.^22^, ^23^ Although, melatonin and EVs play important roles in reproduction, the melatonin and EV‐based communication is absent in embryo culture in vitro. We hypothesized that the absence of this communication may be responsible for the inferior competence of embryos in vitro culture. Thus, we examined the presence of melatonin in oviduct fluid and EVs, after which we attempted to clarify the mechanisms underlying the potential beneficial effects of melatonin and EVs on in vitro embryo development with regard to reactive oxygen species (ROS) levels, epigenetic state, associated gene expression, and developmental competence of embryos.

## 2 MATERIALS AND METHODS

### 2.1 Materials

All chemicals were purchased from Sigma‐Aldrich (St. Louis, MO, USA) unless otherwise indicated.

### 2.2 Animals

White New Zealand rabbits were provided by the Laboratory Animal Centre of Xi'an Jiaotong University Health Science Centre (Xi'an, China). The rabbits were approximately 6 months old and weighed ~3.5 kg. The rabbits were housed under a temperature of 24 ± 1°C, relative humidity of 55% ± 5%, and a photoperiod of 12 hours light:12 hours dark. The animals had access to water and food ad libitum. The experimental protocol was carried out in accordance with the National Institutes of Health Guide for Care and Use of Laboratory Animals and was approved by the Laboratory Animal Care Committee of Xi'an Jiaotong University. Every effort was made to minimize animal pain and suffering and to reduce the number of animals used.

### 2.3 Collection of oviduct fluid, embryos, and serum

Female rabbits were induced with hormones to superovulate. To collect fertilized eggs, we administered 80 IU of PMSG at 9:00 am on day 1. After 72 hours, 100 IU of hCG was administered via intravenous injection, which was immediately followed by mating female rabbits with males. Oviduct fluid, embryos, and serum were collected at 20 hours postmating (5:00 am on day 4). After female rabbits were anesthetized and euthanized, oviduct tubes and uteri were removed from donor rabbits. Oviduct fluid was collected using a plastic pipette (2 mm diameter) connected to a 1‐mL syringe, followed by transfer into a 1‐mL centrifuge tube and incubation for 5 minutes. The fluid was then centrifuged for 10 minutes at 1500 *g*. The supernatant was removed and stored in the dark for further experiments. For embryo collection, the precipitate was resuspended in Dulbecco's phosphate‐buffered saline (DPBS) and embryos collected with a glass needle under a stereomicroscope. Serum was collected by centrifuging the blood samples, after which the supernatant was stored in the dark for subsequent experiments.

### 2.4 Ultracentrifugation, transmission electron microscopy (TEM), and nanoparticle tracking analysis for EVs

EVs were isolated, purified, and identified as previously described.^24^ Briefly, serum or oviduct fluid was collected and ultracentrifuged at 4°C as follows, 300 *g* for 10 minutes, 2000 *g* for 10 minutes, 10 000 *g* for 30 minutes, and 100 000 *g* for 70 minutes. The pellets were retained and resuspended in phosphate‐buffered saline (PBS) and centrifuged at 100 000 *g* for 70 minutes. EVs were obtained as pellets, which were then resuspended in 20 µL PBS for EV visualization using TEM (JEOL). The samples were loaded onto 300‐mesh grids and dried, after which the grids were stained with 2% phosphotungstic acid and imaged. Nanoparticle tracking analysis using NanoSight LM‐10 (Malvern) was performed to determine the size, distribution, and number of particles within pellets according to the manufacturer's instructions. EV collection for embryo culture and melatonin analysis was carried out in the dark.

### 2.5 Western blotting

Western blotting was performed as previously described.^24^ Briefly, RIPA buffer was added to the EV suspensions for membrane protein lysis. Proteins were separated by SDS‐polyacrylamide gel electrophoresis, followed by protein transfer to polyvinylidene difluoride membranes. The membranes were blocked with 5% nonfat milk powder in Tris‐buffered saline containing 0.05% Tween 20 (TBST) for 1 hour. The membrane was then incubated with the CD9 antibody (Santa Cruz Biotechnology), followed by thorough washing in TBST at room temperature (RT). The membrane was then incubated with secondary antibody for 1 hour at RT, followed by washing in TBST. Finally, the blots were developed using an enhanced chemiluminescence kit (Millipore).

### 2.6 In vitro culture of embryos

The collected embryos were washed three times in DPBS and then transferred to synthetic oviductal fluid (SOF) medium in an incubator under 5% (v/v) CO_2_ and 38°C. The embryo culture medium was prepared in a 35‐mm cell culture dish under mineral oil and equilibrated for 2 hours before the embryos were loaded.

### 2.7 EV labeling and internalization by embryos

PKH26 dye was used for fluorescently labeling EVs in this study. Briefly, 120 µL of diluent C was added to 5 µL EVs. PKH26 was then added and incubated for 5 minutes. The reaction was stopped 1 minute after incubation by the addition of an equal volume of 1% bovine serum albumin (BSA)/PBS. Extraction of labeled EVs was performed using the ExoQuick Kit (SystemBio), followed by resuspension in culture medium. Embryos were incubated with the labeled EVs for 24 hours, followed by washing in PBS. The embryos were stained with 4ʹ,6‐diamidino‐2‐phenylindole (DAPI) for 5 minutes before washing in PBS. The samples were then mounted on a coverslip and imaged using a microscope coupled to a digital camera (Nikon). Experiments with the control groups, including blank EVs and blank PKH26, were performed in parallel.

### 2.8 TEM of embryos

Embryos were immobilized in 2.5% glutaraldehyde at 4°C for 2 hours, followed by washes in PBS. The embryos were then fixed with 1% osmium acid for 1 hour and washed in PBS, followed by transfer into preheated 3% agarose solution and centrifugation at 2000 *g* for 1 minute. The embryos were embedded in coagulated agarose and then dehydrated in 30%, 50%, 70%, 85%, 90%, and 95% ethanol for 20 minutes per step, followed by a final step in 100% ethanol. The samples were then treated twice in 100% isoamyl acetate for 15 minutes and then in a solution containing acetone and Epon 812 epoxy resin. Finally, samples were sectioned into ultrathin slices, followed by TEM imaging (JEOL).

### 2.9 Melatonin concentration measurement

The extraction procedure of melatonin was performed as previously described.^25^, ^26^ Briefly, C18 reversed phase columns were pretreated by washing twice with 1 mL of pure methanol and then twice with 1 mL distilled water. Next, 500 μL of sample (serum, oviduct fluid, or embryo culture medium) was added and the columns washed with 1 mL of aqueous methanol (methanol/water, 10/90 by vol). Melatonin was eluted from the columns by adding 2 mL of pure methanol, followed by evaporation to dryness and reconstitution with 500 μL distilled water as previously described.^27^ EVs were mixed with 2 mL methanol and centrifuged at 100 000 *g* at 4°C for 70 minutes. The supernatant was transferred to another tube, evaporated to dryness, and reconstituted with distilled water equal to sample volume. Melatonin levels were measured using an ELISA Kit (Biovision); standards and samples were incubated in duplicate wells of microliter plates. Next, 50 μL biotin‐labeled antibody working solution was added to each 50 μL sample, followed by gentle mixing of the seal‐covered plate. The samples were incubated for 45 minutes at 37°C. After three washes in wash solution, 0.1 mL working solution was added to each well, followed by incubation at 37°C for 30 minutes and washes in wash solution. Subsequently, 90 μL TMB substrate was added to each well, followed by incubation at 37°C in dark for 30 minutes. The reaction was stopped by the addition 50 μL stop solution to each well. The absorbance (OD) was then read by a standard microtiter plate reader at 450 nm. Spike‐and‐recovery experiments were performed to measure melatonin levels in the samples to assess ELISA accuracy. Briefly, a known amount of melatonin was added into the test sample matrix. Then, ELISA was performed to measure melatonin recovery. Recovery was calculated using the formula, recovery (%) = (observed amount−original amount)/spiked amount × 100%. All steps were carried out in the dark.

### 2.10 ROS level determination in embryos

The Reactive Oxygen Species Assay Kit (Beyotime) was used to measure ROS levels. Embryos in each group (n = 15‐20) were incubated in serum‐free culture medium containing 10 mmol/L dichlorodihydrofluorescein diacetate (DCHF‐DA) at 37°C for 20 minutes. The embryos were then washed three times in serum‐free culture medium and imaged using a fluorescence microscope equipped with a digital camera (Nikon), followed by analysis using Image‐Pro Plus (v6.0; Media Cybernetics). The fluorescence intensity value for each embryo was measured using the mean gray values of the area selected by the threshold (MGT), the average of the mean gray values of backgrounds (MB), and the total area of the embryo (TA). The following formula was used to calculate ROS levels, fluorescence intensity = (MGT−MB)/TA.

### 2.11 Apoptosis assay

Apoptosis assays were performed using the DeadEnd Fluorometric TUNEL System (Promega) as previously described.^28^ Briefly, embryos (n = 15‐20) were fixed in 4% paraformaldehyde for 2 hours at RT, permeabilized with 0.5% Triton X‐100 for 5 minutes at RT, and incubated in FITC‐conjugated dUTP and terminal deoxynucleotidyl transferase at 37°C in the dark for 1 hour. The end‐labeling reaction was terminated using 2X SSC in the dark at RT for 15 minutes. Embryos were then incubated with PBS containing 25 µg/mL RNase A in the dark at RT for 30 minutes. After staining with DAPI and washing with PBS in the dark, blastocysts were mounted on a slide with a coverslip. Subsequently, the samples were imaged using a fluorescence microscope equipped with a digital camera (Nikon) and the images analyzed using Image‐Pro Plus (v6.0; Media Cybernetic). The apoptosis index was calculated as the TUNEL‐positive cell number divided by the total cell number of the blastocyst.

### 2.12 Reverse transcription real‐time PCR

Total RNA was extracted from embryos using the Cells‐to‐Signal Kit (Invitrogen) according to manufacturer's instructions.^24^ cDNA was synthesized using the PrimeScript^TM^ RT Reagent Kit (TaKaRa Bio) and real‐time PCR was performed on a CFX96 Real‐Time PCR Detection System (Bio‐Rad Laboratories) with SYBR Premix Ex Taq^TM^ II (TaKaRa Bio). The cycling conditions consisted of initial denaturation at 95°C for 1 minute, followed by 40 cycles of denaturation at 95°C for 5 seconds and primer annealing and extension at 60°C for 30 seconds, after which melting curves were generated by heating from 65 to 95°C. Primer sequences are listed in Table S1. A total of 20 embryos per group were prepared for each reaction in triplicate.

### 2.13 Immunofluorescence

Immunofluorescence staining was performed as previously described.^29^ Embryos were incubated in pronase to remove the zona pellucida and mucin coat. For 5‐methylcytosine (5‐mC) immunostaining, embryos were fixed in 4% paraformaldehyde/PBS for 30 minutes, incubated in 4 mol/L HCl solution for 10 minutes, and neutralized in Tris‐HCl solution (pH 8.0) for 10 minutes. Following a second fixation in 4% paraformaldehyde/PBS, embryos were permeabilized with 0.1% Triton X‐100 for 20 minutes. After blocking overnight at 4°C in 1% BSA solution, samples were stained overnight at 4°C with anti‐5‐mC antibody (Eurogentec). After incubation, embryos were washed in PBS before incubating for 1 hour with Alexa Fluor 488‐labeled secondary antibody (Beyotime) at RT. After secondary antibody staining and washing, embryos were counterstained with DAPI for 30 minutes. For melatonin receptor (MT1) immunostaining, embryos were washed three times before fixation in 4% paraformaldehyde/PBS for 30 minutes at RT. Embryos were then permeabilized with 1% Triton X‐100 in PBS and incubated in blocking solution for 2 hour at RT. Subsequently, the embryos were incubated overnight at 4°C with anti‐MT1 antibody (ABclonal Technology), followed by washing in PBS and incubation with Alexa Fluor 555‐labeled secondary antibody (Beyotime) at RT for 1 hour. Finally, the embryos were counterstained with DAPI for 10 minutes. All samples were mounted on slides using Antifade Mounting Medium (Beyotime) for indirect immunofluorescence imaging under a Nikon Eclipse Ti‐S fluorescence microscope. For inner cell mass (ICM)/trophectoderm (TE) staining, we used an anti‐CDX2 primary antibody (BioGenex). The ICM/TE index was calculated by dividing the cell number of ICM by that of the TE; the ICM cell number was the total cell number minus CDX2‐positive cells, while the TE cell number consisted of the CDX2‐positive cells.

### 2.14 Ammonium assay

Ammonium levels in the embryo culture medium were determined as previously reported.^24^, ^30^ The ultramicrofluorometric technique was used, which is based on the following reaction: glutamate dehydrogenase α‐ketoglutarate + NADH + NH4^+^ → glutamate + NAD^+^ + H_2_O. The reaction reagents consisted of 0.24 mmol/L NADH, 0.75 mmol/L NaHCO_3_, 0.63 mmol/L ADP, 14.15 mmol/L α‐ketoglutarate, and 3 U/mL glutamate dehydrogenase in 157 mmol/L triethanolamine buffer.

### 2.15 Statistical analysis

All experiments were performed in triplicate. Blastocyst rate was defined as the number of blastocysts divided by number of cleaved embryos, and blastocyst rates were analyzed using the chi‐square test. Differences in the relative expression levels of ROS and 5‐mC, apoptotic indexes, ICM/TE ratios, and gene expression were determined by ANOVA. Statistical analysis was conducted using SPSS software (IBM). *P* < .05 were considered statistically significant.

## 3 RESULTS

**Figure 1:**
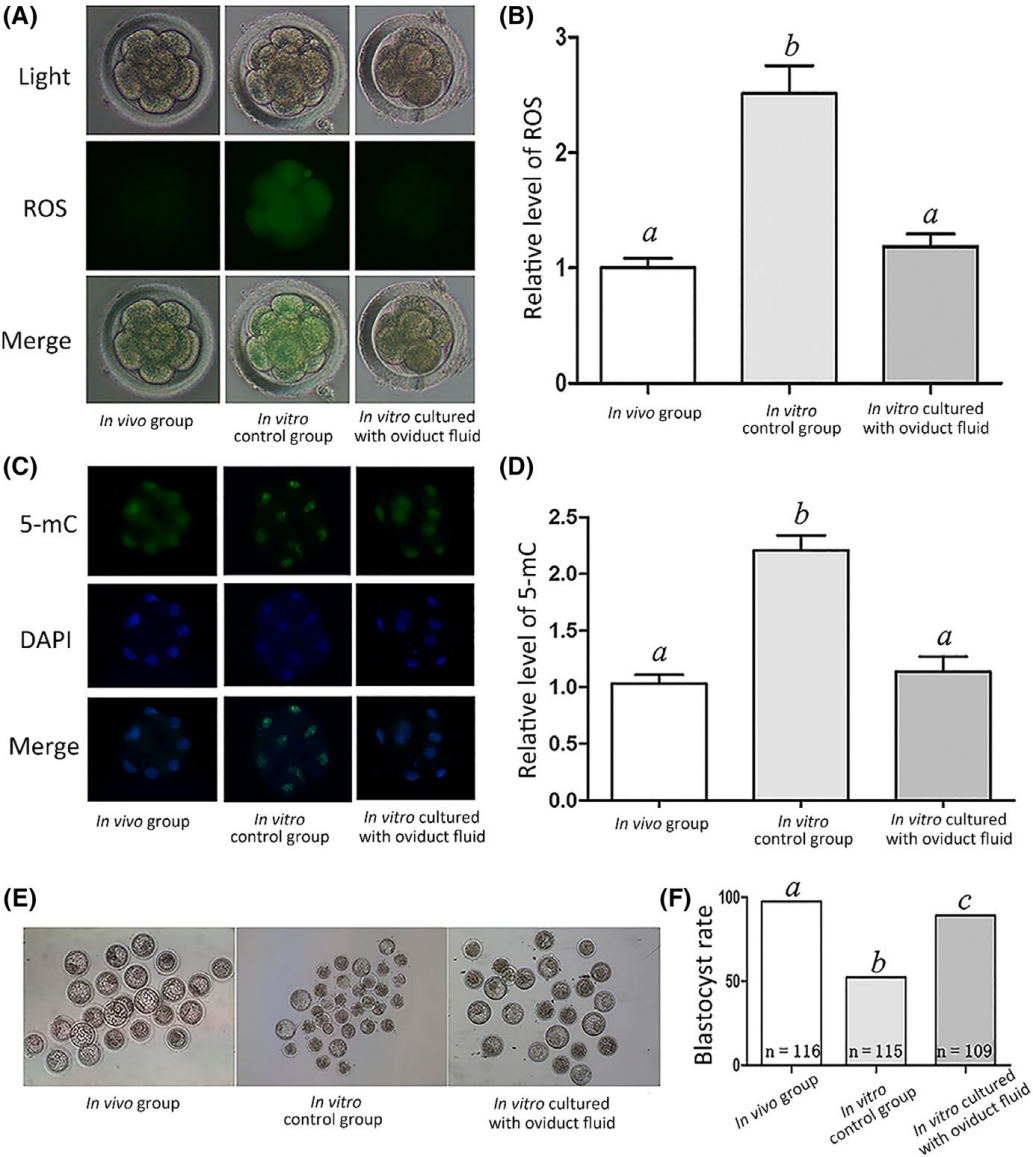
Effect of oviduct fluid on relative ROS and 5‐mC levels at the 8‐cell stage and blastocyst rate. A, ROS staining in embryos. Upper panel: bright field; middle panel: green fluorescence indicating ROS; lower panel: merged bright field and green fluorescence. B, Quantification of ROS fluorescence intensity. C, Staining pattern for 5‐mC in embryos. Green: 5‐mC; blue: DNA. D, Relative fluorescence intensity. E, Representative photographs of embryos on day 3. F, Blastocyst rate on day 3. Different letters above the bars indicate significant differences (*P* < .05)

Embryos at the 8‐cell stage in the in vivo, in vitro control, and in vitro cultures with oviduct fluid groups were analyzed to determine changes in ROS and 5‐mC levels. ROS and 5‐mC levels in the in vitro control group were significantly higher than those of the other two groups (Figure 1A‐D). Moreover, the blastocyst rate in the in vitro control group was significantly lower than that of the other two groups (Figure 1E,1F). The melatonin concentration in serum, oviduct fluid, and culture medium were detected, respectively. The recovery percentage of melatonin ranged from 94.0% to 100.7% in the serum and 93.3% to 98.0% in the oviduct fluid (Table S2); the mean concentrations were 13.1 ± 3.2 and 34.3 ± 5.8 pg/mL in the serum and oviduct fluid, respectively (Table 1). No melatonin was detected in the culture medium.

**Table 1:** The level of melatonin in serum and oviduct fluid

Samples	Serum	Oviduct fluid
Concentration (mean ± SD, pg/mL)	13.1 ± 3.2	34.3 ± 5.8
Maximum concentration (pg/mL)	19.6	44.3
Minimum concentration (pg/mL)	7.9	21.9

**Figure 2:**
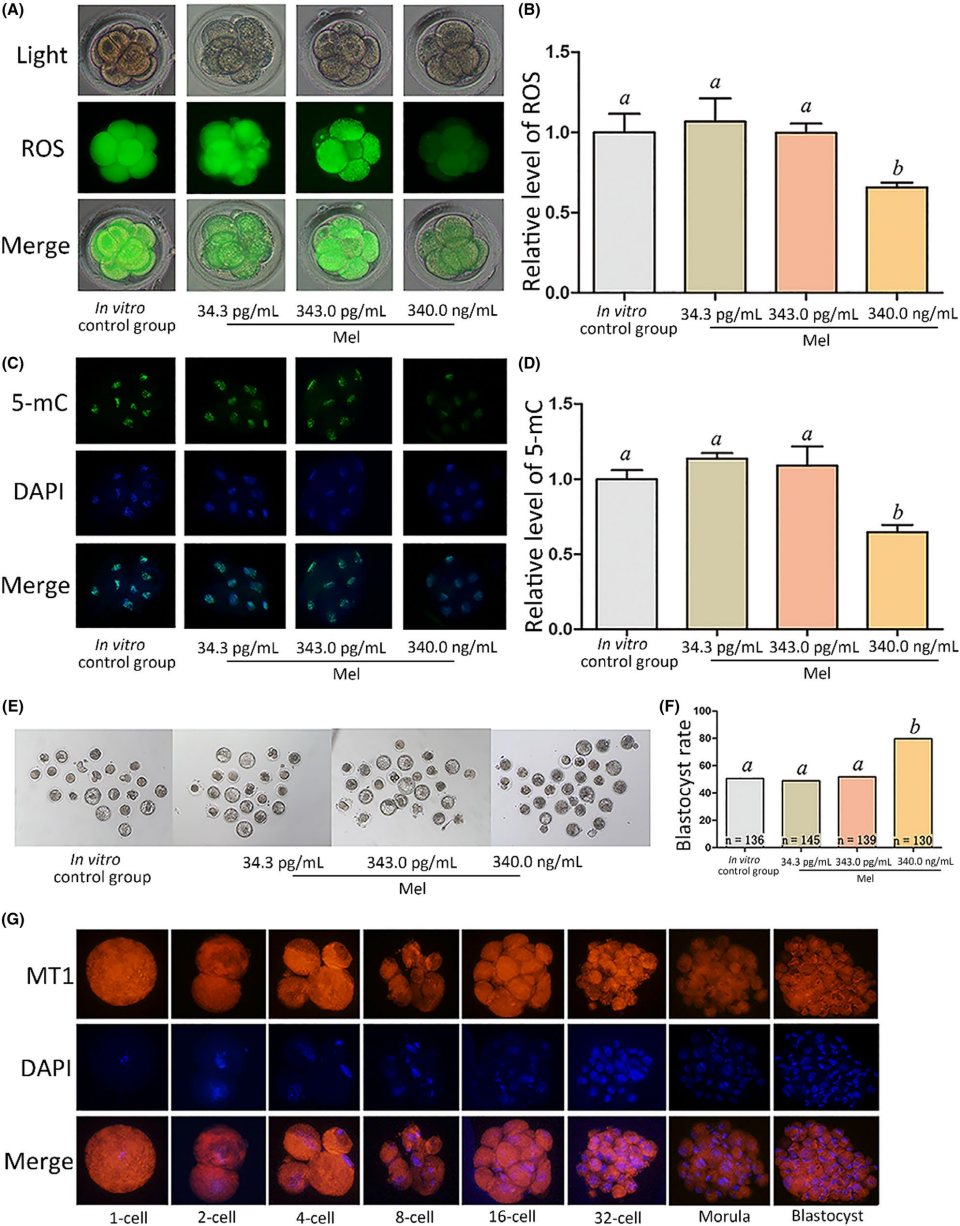
Effect of melatonin on relative ROS and 5‐mC levels at the 8‐cell stage and blastocyst rate. A, ROS staining in embryos. Upper panel: bright field; middle panel: green fluorescence indicating ROS; lower panel: merged bright field and green fluorescence. B, Quantification of ROS fluorescence intensity. C, Staining pattern for 5‐mC in embryos. Green: 5‐mC; blue: DNA. D, Relative fluorescence intensity. E, Representative photographs of embryos on day 3. F, Blastocyst rate on day 3. G, Immunofluorescence of melatonin receptor 1 (MT1) expression at the indicated stages. Orange: MT1; blue: DNA. Different letters above the bars indicate significant differences (*P* < .05). Mel, melatonin

**Figure 3:**
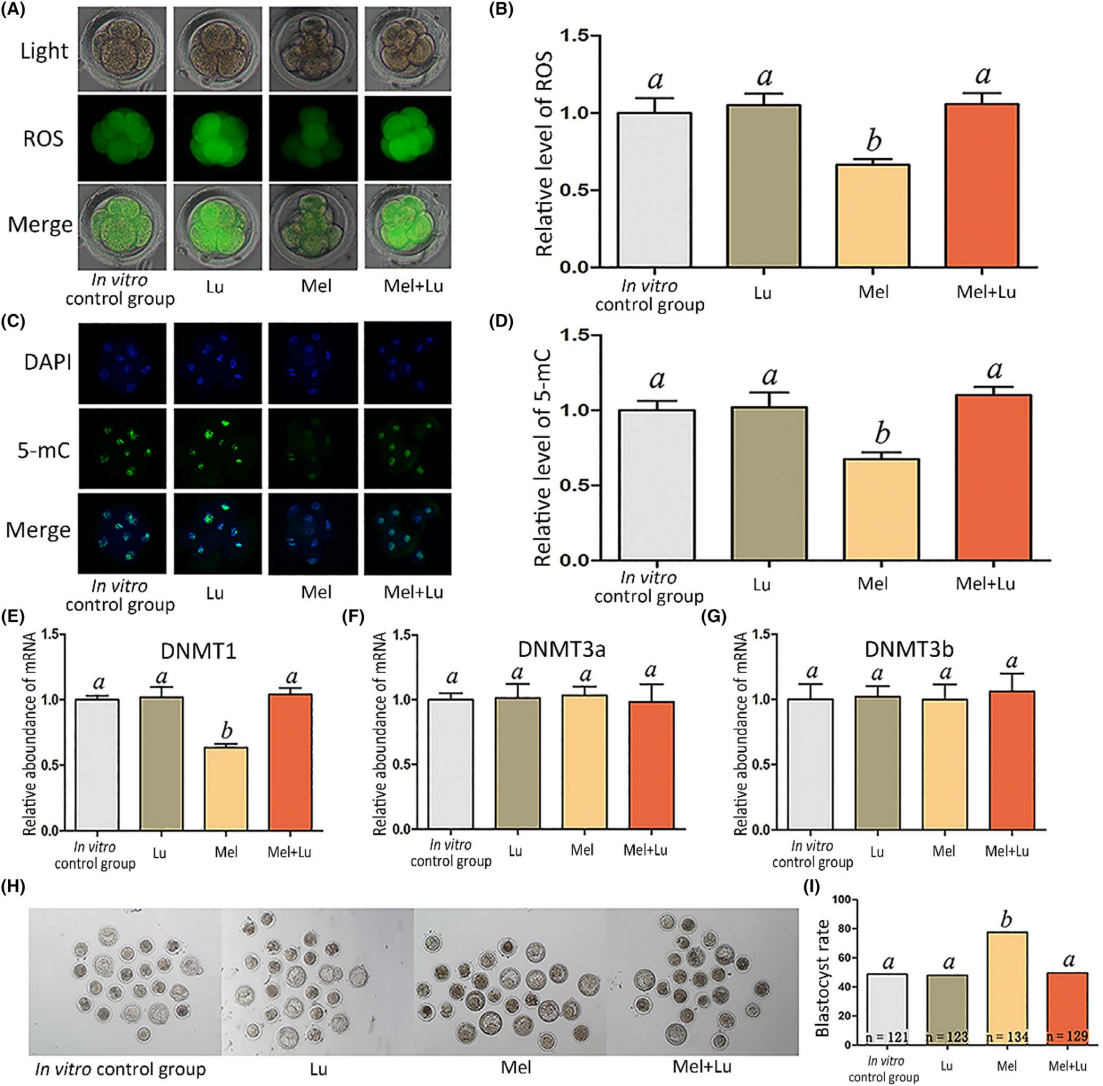
Relative level of ROS and 5‐mC, and epigenetic‐related genes expression at the 8‐cell stage, and blastocyst rate. The treatment dose of melatonin and luzindole was 343.0 and 420.0 ng/mL, respectively. A, ROS staining in embryos. Upper panel: bright field; middle panel: green indicating ROS; lower panel: merged bright field and green fluorescence. B, Quantification of ROS fluorescence intensity. C, Staining pattern for 5‐mC in embryos. Green: 5‐mC; blue: DNA. D, Relative fluorescence intensity. E‐G, Relative mRNA expression levels of DNMT1, DNMT3a, and DNMT3b. H, Representative photographs of embryos on day 3. I, Blastocyst rate on day 3. Different letters above the bars indicate significant differences (*P* < .05). Mel, melatonin; Lu, luzindole

We further examined ROS and 5‐mC levels in embryo in vitro cultures supplemented with different concentrations of melatonin (0, 34.3, pg/mL and 343.0 pg/mL and 343.0 ng/mL) at the 8‐cell stage. There were no significant differences in ROS and 5‐mC levels and blastocyst rate among the groups supplemented with 0, 34.3, or 343.0 pg/mL melatonin (Figure 2A‐F). However, melatonin at a concentration of 343.0 ng/mL significantly decreased ROS and 5‐mC levels and significantly increased blastocyst rate (Figure 2A‐F). Moreover, MT1 expression was detected via immunostaining throughout the preimplantation period (Figure 2G). Next, we used luzindole, a melatonin receptor antagonist, to block melatonin function; compared with the melatonin treatment group, embryos treated with melatonin and luzindole exhibited significantly high levels of ROS, 5‐mC, and DNMT1 (Figure 3A‐E) as well as a lower blastocyst rate (Figure 3H,I). However, DNMT3a and DNMT3b expression levels were not significantly different between the groups (Figure 3F,G). We analyzed DNMT1, DNMT3a, and DNMT3b expression as they are methylation‐associated genes.

**Figure 4:**
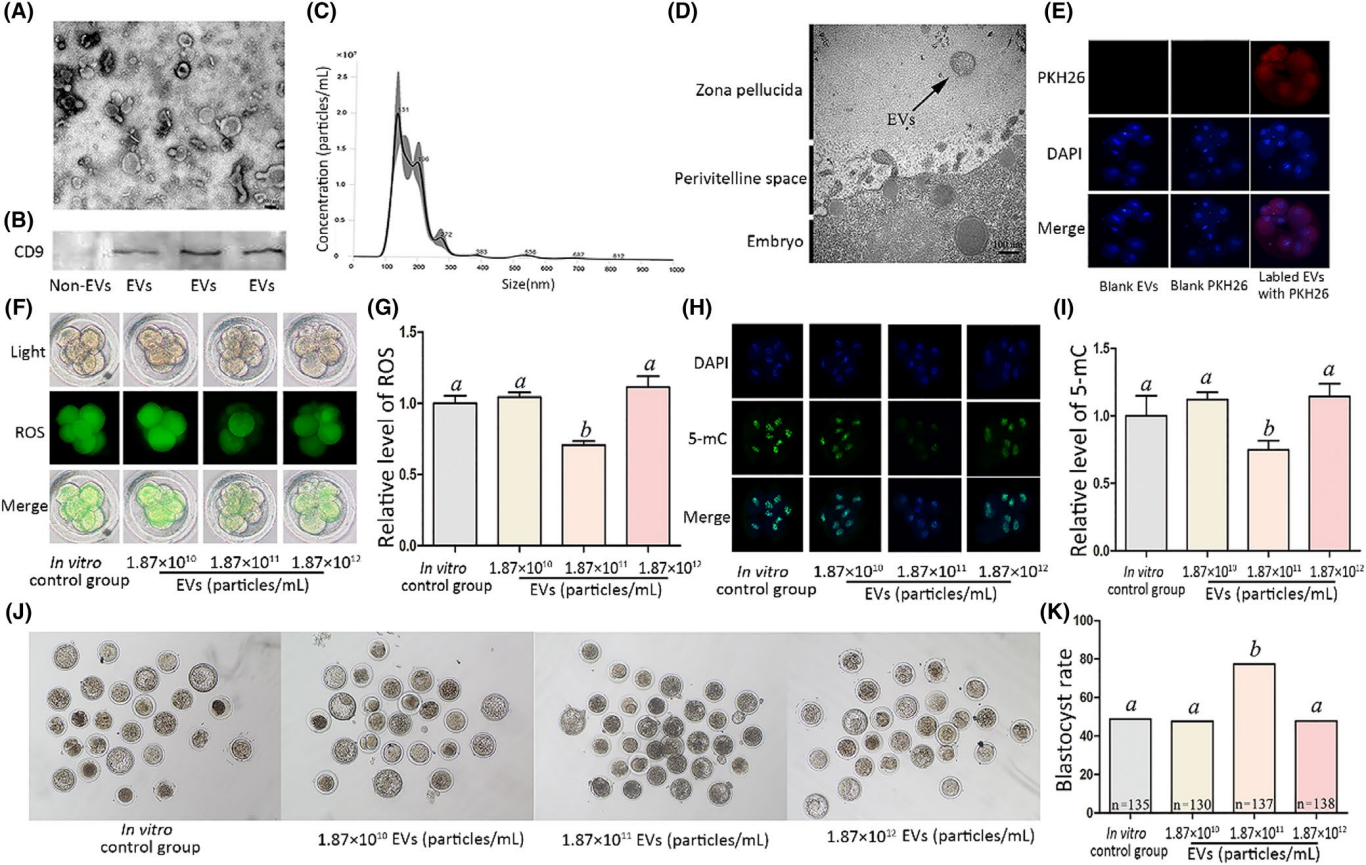
Effect of EVs derived from oviduct fluid on relative ROS and 5‐mC levels at the 8‐cell stage and blastocyst rate. A, Particles were identified using transmission electron microscopy (TEM). B, Western blotting. C, Quantification and size distribution of particles determined by nanoparticle tracking analysis. D, EVs seen through the zona pellucida via TEM. E, Characterization of EVs derived from oviduct fluid interaction with cultured embryos. PKH26 (red): oviduct fluid‐derived EVs; DAPI (blue): nuclei. F, ROS staining in embryos. Upper panel: bright field; middle panel: green fluorescence indicating ROS; lower panel: merged bright field and green fluorescence. G, Quantification of ROS fluorescence intensity. H, Staining pattern for 5‐mC in embryos. Green: 5‐mC; blue: DNA. I, Relative fluorescence intensity. J, Representative photographs of embryos on day 3. K, Blastocyst rate on day 3. Scale bar = 100 nm. Different letters above the bars indicate significant differences (*P* < .05). EVs, extracellular vesicles

EVs were isolated from oviduct fluid for TEM and further identified by Western blotting and nanoparticle tracking analysis. The particles exhibited typical vesicle structure and CD9 signals were detected in those extracted from oviduct fluid (Figure 4A,4B). The concentration of EVs derived from oviduct fluid was 1.87 × 10^10^ ± 1.37 × 10^9^ particles/mL (Figure 4C). TEM of the embryos revealed that EVs were detected throughout the zona pellucida (ZP), indicating that EVs can cross the ZP (Figure 4D). Immunostaining of oviduct fluid‐isolated EVs with the membrane dye PKH26 demonstrated fluorescent signals along the embryo surface, whereas no signals were detected for the control groups (Figure 4E), indicating a physical interaction between oviduct fluid‐derived EVs and embryos. Next, we added different quantities of oviduct fluid‐derived EVs (0, 1.87 × 10^10^, 1.87 × 10^11^, and 1.87 × 10^12^ particles/mL) to the embryo culture. At a concentration of 1.87 × 10^11^ particles/mL, ROS and 5‐mC levels were significantly lower, while the blastocyst rate was significantly higher than those of the other groups (Figure 4F‐K). It is worth noting that ROS and 5mC levels were higher at a concentration of 1.87 × 10^12^ particles/mL than at 1.87 × 10^11^ particles/mL. To answer why, we determined the ammonium concentration of the cultured medium of each group and found that it was over 300 μmol/L at 24, 48, and 72 hours after culturing under a concentration of 1.87 × 10^12^ particles/mL EVs (Table S3).

**Figure 5:**
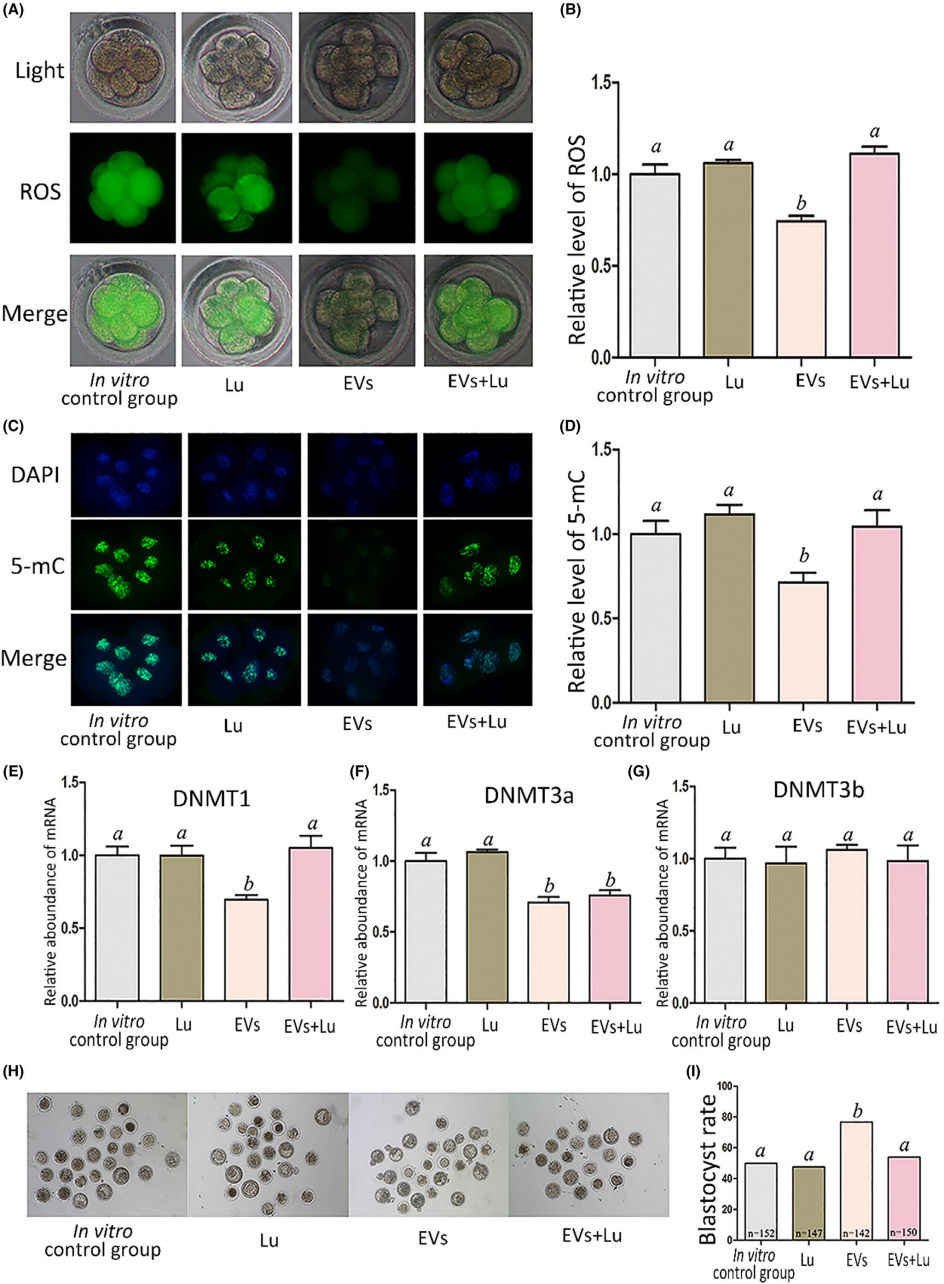
Effect of EVs and luzindole on relative ROS and 5‐mC levels and epigenetic‐related gene expression at the 8‐cell stage as well as blastocyst rate. Treatment doses of EVs and luzindole were 1.87 × 10^11^ particles/mL and 420.0 ng/mL, respectively. A, ROS staining in embryos. Upper panel: bright field; middle panel: green fluorescence indicating ROS; lower panel: merged bright field and green fluorescence. B, Quantification of ROS fluorescence intensity. C, Staining for 5‐mC in embryos. Green: 5‐mC; blue: DNA. D, Relative fluorescence intensity. E‐G, Relative mRNA expression levels of DNMT1, DNMT3a, and DNMT3b. H, Representative photographs of embryos on day 3. I, Blastocyst rate on day 3. Different letters above the bars indicate significant differences (*P* < .05). EVs, extracellular vesicles; Lu, luzindole

The concentration of melatonin in oviduct fluid‐derived EVs was then examined. We found a melatonin recovery rate of 94.6%‐100.7% (Table S4); the spike‐and‐recovery experiment results indicated that the associated methods applied in this study are reliable. The mean concentration of melatonin in these EVs was 43.4 ± 4.2 pg/mL (Table 2). To determine whether EV function is associated with melatonin, we added luzindole to the EVs treatment. Embryos treated with EVs and luzindole showed higher levels of ROS, 5‐mC, DNMT1 expression, and lower blastocyst rates compared with those in the EV only treatment group (Figure 5A‐E,H,I). Meanwhile, DNMT3a expression levels were significantly lower in all EV groups, regardless of luzindole treatment, than in the others (Figure 5F). Additionally, there were no significant differences in DNMT3b expression levels among the groups (Figure 5G).

**Table 2:** The level of melatonin in EVs derived from serum and EVs derived from oviduct fluid

Samples	EVs derived from serum	EVs derived from oviduct fluid
Concentration (mean ± SD, pg/mL)	12.7 ± 2.6	43.4 ± 4.2
Maximum concentration (pg/mL)	15.1	52.3
Minimum concentration (pg/mL)	8.9	29.5

**Figure 6:**
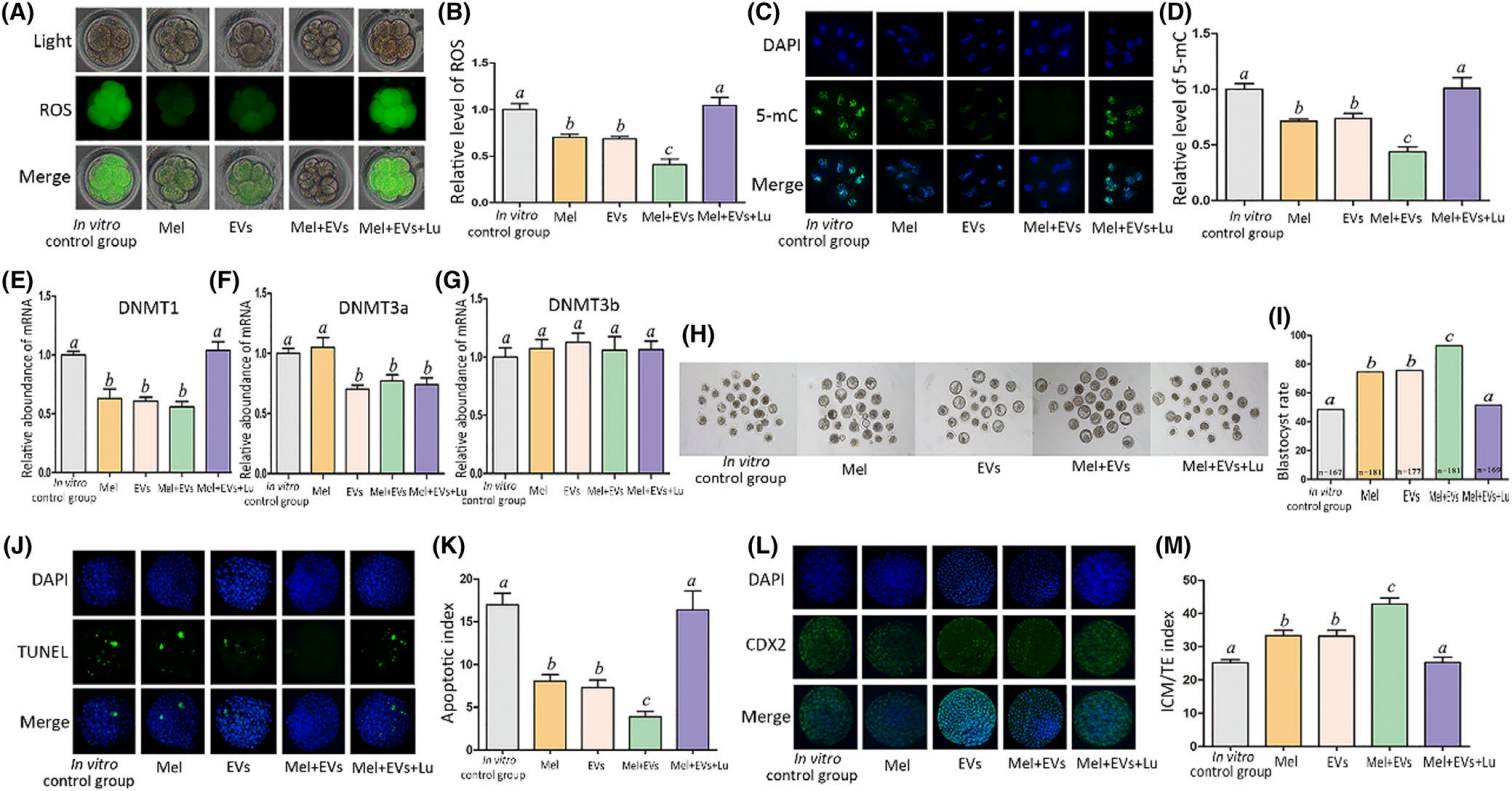
Effect of the EVs and melatonin combination treatment on relative ROS and 5‐mC levels and epigenetic‐related gene expression at the 8‐cell stage as well as blastocyst rate, apoptosis index, and ICM/TE index. The groups were in vitro control group, melatonin group (343.0 ng/mL), EVs (1.87 × 10^11^ particles/mL) group, EVs (1.87 × 10^10^ particles/mL) + Mel (34.3 pg/mL) group, and EVs (1.87 × 10^10^ particles/mL) + Mel (34.3 pg/mL) + Lu (420.0 ng/mL) group. A, ROS staining in embryos. Upper panel: bright field; middle panel: green fluorescence indicating ROS; lower panel: merged bright field and green fluorescence. B, Quantification of ROS fluorescence intensity. C, Staining pattern for 5‐mC in embryo. Green: 5‐mC; blue: DNA. D, Relative fluorescence intensity. E‐G, Relative mRNA expression levels of DNMT1, DNMT3a, and DNMT3b. H, Representative photographs of embryos on day 3. I, Blastocyst rate on day 3. J, TUNEL assay of apoptotic blastomeres. DAPI (blue): DNA. K, Apoptosis index of blastocysts. L, Immunostaining for TE (CDX2 antibody, green) and blastomeres (DAPI, blue). M, ICM/TE index of blastocysts. Different letters above the bars indicate significant differences (*P* < .05). EVs, extracellular vesicles; Lu, luzindole; Mel, melatonin; ICM, inner cell mass; TE, trophectoderm

Finally, we combined EVs (1.87 × 10^10^ particles/mL) and melatonin (34.3 pg/mL) to test their effects on embryo culture. The combination significantly decreased ROS and 5‐mC levels (Figure 6A‐D). DNMT1 expression levels were higher in the in vitro control group and EV + melatonin + luzindole group than in other groups (Figure 6E), while DNMT3a levels were higher in the in vitro control group and melatonin‐treated group than in other groups (Figure 6F). However, DNMT3b expression levels were not significantly different between the groups (Figure 6G). The combination of EVs and melatonin significantly increased the blastocyst rate, while the apoptosis and ICM/TE indexes of blastocysts were significantly lower and significantly higher than that of other groups, respectively (Figure 6H‐M). Luzindole addition effectively blocked the positive combined effect of EVs and melatonin in term of the ROS, 5‐mC, DNMT1 expression, blastocyst rate, the apoptosis and ICM/TE indexes (Figure 6A‐E, H‐M).

## 4 DISCUSSION

Recent research has highlighted that oviduct fluid plays an important role in the fertilization and development of preimplantation embryos.^31^ Embryos cultured in vitro display higher ROS levels, abnormal epigenetic changes, and disordered gene transcription due to the loss of regulators derived from oviduct fluid.^32^ Normal embryonic development is disrupted under extremely high ROS levels, which can lead to mitochondria, cell membrane, and DNA damage as well abnormal gene expression.^33^ In this study, embryos cultured in vitro control group exhibited higher ROS levels than those in in vivo, which is consistent with a previous report.^34^ Notably, we found that embryos cultured in oviduct fluid in vitro exhibited relatively lower levels of ROS than those cultured in vitro control group, suggesting that oviduct fluid has an antioxidant capacity.

During the preimplantation stage of embryos, paternal and maternal genomes undergo dramatic reprogramming changes,^35^ particularly embryonic genome activation (EGA).^36^ EGA of rabbit embryos generally occurs at the 8‐cell stage and is characterized by strict temporal and stage‐specific gene regulation.^32^, ^37^ In particular, DNA methylation, an important epigenetic modification, plays a crucial role in regulating gene expression and other biological processes.^38^ DNA methylation states are altered by in vitro culture conditions of preimplantation stage rabbit embryos.^32^ In agreement with a previous study,^34^ we found that embryos cultured in vitro control group exhibited higher 5‐mC levels than those in vivo. When embryos were cultured in oviduct fluid in vitro, we observed relatively lower 5‐mC levels than those of embryos cultured in vitro control group, indicating that oviduct fluid plays an important role in embryonic epigenetic modifications.

Melatonin in serum is mainly derived from the pineal gland.^8^ Other organs such as the ovary can produce melatonin, which plays a crucial role in oocyte maturation.^39^, ^40^ Inspired by this, we examined melatonin expression and found it present in oviduct fluid at a concentration similar to that in follicular fluid.^41^, ^42^ Our results indicate that melatonin can rectify the abnormal levels of 5‐mC, potentially in a DNMT1‐related manner; downregulation of DNMT1, ROS, and 5‐mC levels and increase in blastocyst rate by melatonin; and the attenuation of these effects by luzindole.

The effective dosage of melatonin in vitro was higher than its concentration in vivo; thus, we speculated that other important factors, such as EVs in oviduct fluid, are positively associated with melatonin function. In our previous study, we found that embryos can secrete EVs, which are essential for blastocyst formation and paracrine communication.^24^, ^43^ In this study, we found that EVs exist in the ZP—consistent with a previous study^44^—and that EVs derived from oviduct fluid are distributed on the surface of embryos, suggesting a physical interaction between EVs and embryos. Notably, oviduct fluid‐derived EVs decreased ROS, 5‐mC, DNMT1, and DNMT3a levels and increased the blastocyst rate of embryos in the in vitro experiments. This is in line with a previous study where EVs derived from bovine oviduct fluid were found crucial to fertilization and early‐stage embryonic development.^22^ Moreover, this beneficial effect of EVs was attenuated by luzindole in our study, indicating a positive association between melatonin and EVs on ROS and 5‐mC levels. However, as luzindole did not affect DNMT3a expression in EVs, we believe that other factors may be involved. Interestingly, EVs at a concentration of 1.87 × 10^12^ particles/mL led to higher ROS and 5‐mC levels than did a concentration of 1.87 × 10^11^ particles/mL. This may be because at increasing EV concentrations, more ammonium is produced by specific proteins or polypeptides and accumulates.^24^ Indeed, at the higher EV concentration, ammonium concentrations were over 300 μmol/L in cultured media after 24 hours. Embryos exposed to 300 μmol/L ammonium induce aberrant blastocyst differentiation, gene expression, and fetal development.^30^ Thus, an excessive dose of EVs may have adverse effects on embryos.

EVs consist of lipids, proteins, RNA, and other components.^45^ EV lipidomic studies characterized EV lipid species from reticulocytes,^46^ B lymphocytes,^47^ mast cells,^48^ dendritic cells, and prostate cancer cells,^49^ and approximately 280 molecular lipid species were characterized from prostate cancer cellline‐derived EVs,^49^ while 319 proteins were identified in EVs,^22^ some of which may bind to melatonin, such as albumin,^50^ and pepsin.^51^ Melatonin can interact with hydrophobic sites due to its lipid solubility.^52^ Moreover, the cellular permeability of melatonin is affected by different molecules, such as complex formation with chromogranin and ATP.^53^ Melatonin may also be retained in vesicles.^53^ In this study, we found that melatonin is abundantly present in EVs. We thus speculate that EVs are associated with melatonin retention and transport,^22^ possibly providing a stable environment for melatonin as a protective mechanism, or that specific EV proteins play an important regulatory role in melatonin signaling. Melatonin or EVs alone at an equivalent concentration to that in oviduct fluid did not have significant impacts on ROS and 5‐mC levels and blastocyst rate. On the other hand, combining melatonin and EVs at an equivalent concentration to that in oviduct fluid significantly decreased ROS and 5‐mC levels, suggesting a synergistic mechanism of EVs and melatonin on enhancing embryo development. Indeed, luzindole attenuated the positive effects of melatonin + EV treatment, indicating that their synergistic action is melatonin dependent. Moreover, embryos with high developmental competence are characterized by low apoptosis and high ICM/TE indexes, which were reflected in embryos treated with melatonin + EVs; these embryos also had a high blastocyst development rate.

In summary, we characterized the role of EVs and melatonin on embryonic development in vitro and identified their synergistic mechanism of enhancing in vitro embryonic development by regulating ROS and 5‐mC levels. These findings have important implications for assisted reproduction, as they provide new insights into the communication between the embryo and maternal oviduct and a new paradigm for the biomedical application of EVs and melatonin in the future.

## CONFLICT OF INTEREST

The authors declare no conflict of interest.

## AUTHOR CONTRIBUTIONS

Enqi Liu and Pengxiang Qu contributed to experimental design, data interpretation, as well as drafting and critical revision of the manuscript. Pengxiang Qu, Shiwei Luo, Yanru Zhang, Xiaojie Song, Yuchen Li, Xuetao Yuan, and Zujie Lin participated in study design and coordination. Enqi Liu and Yue Du reviewed the article. All authors read and approved the final version.
