# Supplementary material for: Extracellular vesicles and melatonin benefit embryonic develop by regulating reactive oxygen species and 5‐methylcytosine
Source: J Pineal Res. 2020 Feb 16;68(3):e12635. doi: 10.1111/jpi.12635 (PMC7154726; doi:10.1111/jpi.12635)
Supplement: Supplementary file 1 [file JPI-68-e12635-s001.docx]

**Table S1. Summary of Real-Time PCR primers used.**

| Gene name | Accession | Forward primer sequence (5´‐3´) | Reverse primer sequence (5´‐3´) |
| --- | --- | --- | --- |
| DNMT1 | XM_008250745.2 | TTCCTGCAGAAGAACCGGAC | GCTCGTACTTGAGATCCGGG |
| DNMT3a | XM_017340920.1 | AAGCACATCCAGGAATGGGG | ATGCCAACGGCCTGTTCATA |
| DNMT3b | XM_008256121.2 | AAGCCGCCCAAGTAAACGTA | CGTTCTGGTCTCACGGAACA |
| HPRT1 | NM_001105671.1 | ACGTCGAGGACTTGGAAAGGGTGTT | GGCCTCCCATCTCCTTCATCACATC |
| H2A.Z | XM_017347391.1 | GCTTGTTTGAGCTTCCGCAG | AGTTGCAAATGACGAGGGGT |
