# Supplementary material for: Extracellular vesicles and melatonin benefit embryonic develop by regulating reactive oxygen species and 5‐methylcytosine
Source: J Pineal Res. 2020 Feb 16;68(3):e12635. doi: 10.1111/jpi.12635 (PMC7154726; doi:10.1111/jpi.12635)
Supplement: Supplementary file 2 [file JPI-68-e12635-s002.docx]

**Table S2. Recovery of melatonin for embryo culture medium, serum and oviduct fluid.**

| Sample | Embryo culture medium | | Serum | | Oviduct fuid | |
| --- | --- | --- | --- | --- | --- | --- |
| Added  (pg/mL) | Concentration  (pg/mL) | Recovery | Concentration  (pg/mL) | Recovery | Concentration  (pg/mL) | Recovery |
| Blank | 0 | / | 13.3±0.9 | / | 35.2±1.4 | / |
| 5 | 4.8 | 96.0% | 18.0±1.4 | 94.0% | 40.1±1.2 | 98.0% |
| 15 | 13.9 | 92.7% | 28.4±0.7 | 100.7% | 49.2±0.7 | 93.3% |
| 25 | 24.3 | 97.2% | 37.7±1.4 | 97.6% | 59.4±1.2 | 96.8% |
| 50 | 48.1 | 96.2% | 61.7±1.5 | 96.8% | 83.8±3.6 | 97.2% |
| 100 | 97.2 | 97.2% | 108.7±2.3 | 95.4% | 129.2±3.3 | 94.0% |
