# Supplementary material for: Extracellular vesicles and melatonin benefit embryonic develop by regulating reactive oxygen species and 5‐methylcytosine
Source: J Pineal Res. 2020 Feb 16;68(3):e12635. doi: 10.1111/jpi.12635 (PMC7154726; doi:10.1111/jpi.12635)
Supplement: Supplementary file 3 [file JPI-68-e12635-s003.docx]

**Table S3. Ammonium concentration of cultured medium in different quantities of EVs derived from oviduct fluid.**

| Group(EVs) | 0h | 24h (μM) | 48h(μM) | 72h(μM) |
| --- | --- | --- | --- | --- |
| 0 | 16.5±1.6 | 32.6±2.6 | 82.0±4.9 | 164.5±10.3 |
| 1.87×10^10^ particles/mL | 16.8±0.9 | 37.9±1.3 | 90.7±3.4 | 170.0±12.2 |
| 1.87×10^11^ particles/mL | 17.2±2.1 | 79.6±4.0 | 121.6±5.7 | 210.5±5.2 |
| 1.87×10^12^ particles/mL | 19.2±3.3 | 313.0±11.6* | 412.7±15.4* | 487±13.6* |

^*^The ammonium concentration of cultured medium in the EVs group(at the concentration of 1.87×10^12^ particles/mL) at 24h, 48h and 72h post culture were over 300μM.
