# Supplementary material for: Extracellular vesicles and melatonin benefit embryonic develop by regulating reactive oxygen species and 5‐methylcytosine
Source: J Pineal Res. 2020 Feb 16;68(3):e12635. doi: 10.1111/jpi.12635 (PMC7154726; doi:10.1111/jpi.12635)
Supplement: Supplementary file 4 [file JPI-68-e12635-s004.docx]

**Table S4. Recovery of melatonin for extracellular vesicles(EVs) derived from serum and oviduct fluid.**

| Sample | EVs derived from serum | | EVs derived from oviduct fluid | |
| --- | --- | --- | --- | --- |
| Added  (pg/mL) | Concentration  (pg/mL) | Recovery | Concentration  (pg/mL) | Recovery |
| Blank | 16.1±1.4 | / | 43.5±2.0 | / |
| 5 | 21.2±0.6 | 102.0% | 48.3±1.5 | 96.0% |
| 15 | 31.3±1.4 | 101.3% | 58.1±1.3 | 97.3% |
| 25 | 39.4±1.5 | 93.2% | 67.5±2.5 | 96.0% |
| 50 | 63.4±1.4 | 94.6% | 90.8±4.7 | 94.6% |
| 100 | 112.3±4.6 | 96.2% | 144.2±5.6 | 100.7% |
